# A Novel Image Encryption Algorithm Based on Improved Arnold Transform and Chaotic Pulse-Coupled Neural Network

**DOI:** 10.3390/e24081103

**Published:** 2022-08-10

**Authors:** Jinhong Ye, Xiangyu Deng, Aijia Zhang, Haiyue Yu

**Affiliations:** 1 College of Physics and Electronic Engineering, Northwest Normal University, Lanzhou 730070, China; 2 Engineering Research Center of Gansu Province for Intelligent Information Technology and Application, Lanzhou 730070, China

**Keywords:** chaotic pulse-coupled neural network, Arnold transform, chaotic sequence, image scrambling, image encryption

## Abstract

Information security has become a focal topic in the information and digital age. How to realize secure transmission and the secure storage of image data is a major research focus of information security. Aiming at this hot topic, in order to improve the security of image data transmission, this paper proposes an image encryption algorithm based on improved Arnold transform and a chaotic pulse-coupled neural network. Firstly, the oscillatory reset voltage is introduced into the uncoupled impulse neural network, which makes the uncoupled impulse neural network exhibit chaotic characteristics. The chaotic sequence is generated by multiple iterations of the chaotic pulse-coupled neural network, and then the image is pre-encrypted by XOR operation with the generated chaotic sequence. Secondly, using the improved Arnold transform, the pre-encrypted image is scrambled to further improve the scrambling degree and encryption effect of the pre-encrypted image so as to obtain the final ciphertext image. Finally, the security analysis and experimental simulation of the encrypted image are carried out. The results of quantitative evaluation show that the proposed algorithm has a better encryption effect than the partial encryption algorithm. The algorithm is highly sensitive to keys and plaintexts, has a large key space, and can effectively resist differential attacks and attacks such as noise and clipping.

## 1. Introduction

In the age of informatization and digitization, digital images, as the mainstream carrier of information transmission, play an indispensable role in information transmission and storage. In the era of rapid information flow, how to protect user privacy; ensure the safe transmission and storage of image information; and avoid theft, tampering, and public dissemination by illegal persons in the process of data transmission has become a hot issue in information security research in recent years [1]. The image encryption technology has become one of the effective technical means to solve this problem, which can well ensure the security of the image information and data of the user in the military, aviation, and other fields and will not easily lead to the leakage of information [2].

At present, there are two main technical methods for image encryption—the scrambling of image position and the replacement of image pixel value. Among them, the image scrambling method mainly uses a certain transformation to scramble the position of the image pixel points and then changes the positional relationship between the original image pixel points. This method achieves the effect of image encryption by reducing the correlation between adjacent pixels in the image. Commonly used image position scrambling methods include Zigzag transformation, Arnold transformation (also known as cat mapping), Standard mapping, magic square transformation, etc. For example, Rui Liang et al. [3] proposed a double-scrambling encryption algorithm based on the two-dimensional Arnold transform. The algorithm not only changes the basic statistical information of the image but also changes the texture feature information of the image. Xingyuan Wang et al. [4] proposed an image encryption algorithm based on dynamic line scrambling and Zigzag transform. This algorithm uses the method of different directions of odd and even lines to traverse and scramble the image and proposes a new scrambling method based on the idea of sawtooth scrambling to improve the encryption effect of the image. ChunLai Li et al. [5] proposed a grayscale image encryption technology scheme based on bit-level scrambling and multiplicative diffusion architecture. Firstly, binary tree scrambling, flip scrambling, and improved circular index scrambling are used to achieve scrambling, and then the improved GF (257) field multiplication is used to diffuse the scrambled components to improve the security of image encryption. The advantage of the image position scrambling encryption method is that the method is simple and easy to implement, but this method has certain limitations, so the scrambling effect is not ideal. Therefore, using this method alone for image encryption processing has low complexity and low security.

The image pixel value substitution method mainly uses a chaotic system to generate a chaotic sequence, then uses the generated chaotic sequence to perform XOR processing with the original image to generate a new pixel value, and finally replaces the pixel value of the original image with the new pixel value. This method mainly achieves the effect of encryption by reducing the correlation between image pixels. The commonly used chaotic systems include the logistic map chaotic system, Henon map chaotic system, cellular neural network chaotic system, and Lorentz chaotic system. For example, Noura Khalil et al. [6] proposed an image encryption algorithm based on a chaotic system. This method first uses a chaotic map to scramble the image, then uses the logistic-tent chaotic map to generate a chaotic sequence, and then performs a bitwise XOR operation on the generated chaotic sequence and the scrambled image to obtain a ciphertext image. Xiaohong Gao et al. [7] proposed an image encryption algorithm based on a two-dimensional hyperchaotic map. The algorithm first scrambles the rows and columns of the image and then diffuses the pixel values to obtain an encrypted image. Gopal Ghosh et al. [8,9] proposed a security monitoring framework for IoT systems based on image encryption. The initial parameters of the hyperchaotic map are obtained based on the partially regenerated non-dominated optimization algorithm (PRNDO). The framework then uses the hyperchaotic map to generate pseudo-random sequences. The encrypted image is finally generated through the permutation and diffusion process. Christophe Magloire, Lessouga Etoundi et al. [10] proposed an image encryption method combining the existing chaotic maps to construct a hyper-chaotic map to obtain a composite coupled hyper-chaotic map. Anand B. Joshi et al. [11] proposed an image encryption algorithm based on two-dimensional discrete wavelet transform and three-dimensional logistic chaotic mapping. The algorithm transforms the image in frequency domain and then uses the chaotic map to encrypt and decrypt the image in the frequency domain. Although the encryption method based on a chaotic system has been well improved in encryption security and complexity, most chaotic systems are sensitive to salt and pepper noise, Gaussian noise, and shearing attacks and are prone to avalanche effects. The anti-attack ability of the encrypted image is poor, and the encrypted image is easily changed after a small change or an external attack, which can easily lead to a huge change in the decrypted image, and it is difficult to retain the basic feature information of the original image.

Therefore, this paper proposes a new image encryption algorithm based on improved Arnold transform and chaotic pulse-coupled neural network (PCNN). The algorithm improves the original Arnold transform and solves the limitation that the Arnold transform is only suitable for square images. At the same time, the pixel position index is introduced into the algorithm to solve the periodic problem of Arnold transform, which effectively improves the security of image scrambling by Arnold transform, and it is not easy for illegal persons to decipher by using the periodic characteristics of Arnold transform. In order to further enhance the effect of image encryption and improve the security of the image, this paper also proposes a new encryption method based on a chaotic pulse-coupled neural network. The chaotic pulse-coupled neural network system has many parameters, strong sensitivity to the initial value and large key space, and good encryption performance.

The main contributions of this work include: (1) This paper proposes a new image encryption algorithm based on improved Arnold transform and chaotic pulse-coupled neural network, which has good encryption performance and is safe and reliable. (2) In order to solve the limitation that the traditional Arnold transform can only scramble square image, this paper proposes a variable sliding window Arnold transform method. (3) In order to enhance the security of image encryption, we introduce the pixel position index to increase the scrambling degree of the image and also solve the periodic problem of Arnold transform.

The work arrangement of this paper is as follows. In Section 2, we propose the chaotic PCNN model and image encryption algorithm and introduce its basic principles. In Section 3, the specific scheme and process of image encryption and decryption are introduced. In Section 4, we introduce the experimental environment, parameter settings, and experimental results. In Section 5, the security of image encryption is analyzed, and the performance of the proposed encryption algorithm is evaluated through experiments in the chapter. Finally, the conclusion is given in Section 6.

## 2. Theoretical Analysis of Chaotic PCNN Model

### 2.1. Uncoupled Linking PCNN Model

**Figure 1:**
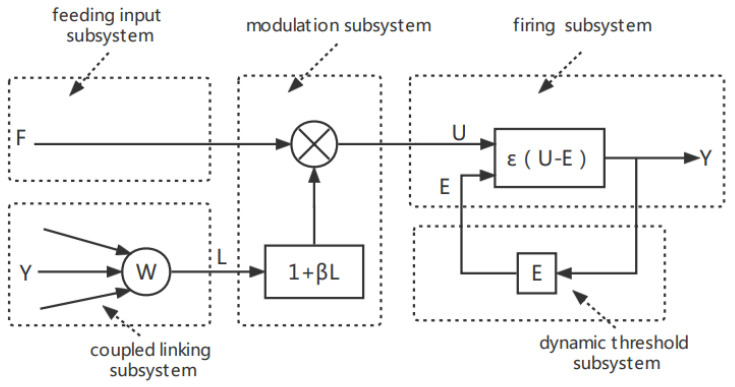
PCNN simplified model.

A pulse-coupled neural network is a single-layer, third-generation artificial neural network that does not require training [12]. In 1990, Eckhorn et al. proposed a neural network model based on the signal transduction of cats’ visual cortices [13]. In 1999, Johnson et al. improved it into a model suitable for image processing and named it PCNN [14]. Up to now, the model has been widely used in various fields of digital image processing and has achieved good results. PCNN is a mathematical model that simulates the relationship between the structure of biological neurons and the interaction between neurons [15], and its simplified model is shown in Figure 1.

PCNN model can be divided into uncoupled link and coupled link. This paper is mainly based on uncoupled linking PCNN. The model can be simplified, as shown in the following Equations (1)–(3):

(1) $$U_{ij}(n)=F_{ij}(n)=S_{ij}+e^{-a_{F}}F_{ij}(n-1)$$

(2) $$E_{ij}(n)=e^{-a_{E}}E_{ij}(n-1)+V_{E}Y_{ij}(n-1)$$

(3) $$Y_{ij}(n)=\varepsilon [U_{ij}(n)-E_{ij}(n)]$$

According to Equations (1)–(3), the iterative equation of uncoupled linking PCNN can be obtained, as shown in Equation (4).

(4) $$Y_{ij}(n)=\varepsilon [S_{ij}+e^{-a_{F}}F_{ij}(n-1)-e^{-a_{E}}E_{ij}(n-1)-V_{E}Y_{ij}(n-1)]$$

Equations (1)–(3) represent the feeding input subsystem, the firing subsystem, and the dynamic threshold subsystem, respectively, where $S_{ij}$ represents the input of the PCNN and is the normalized gray value of a pixel corresponding to a neuron. Subscripts i, j represent the position of the center pixel of the PCNN. $\alpha_{E}$ represents the time constant of the iteration decay of the dynamic threshold subsystem. $V_{E}$ represents the linking weight amplification coefficient between the dynamic threshold and the firing subsystem. $Y_{ij}$, the output of the PCNN, represents the firing state of the neuron (0 or 1). The state of a neuron (i.e., firing or fire extinguishing) depends upon the output of the firing subsystem.

#### 2.1.1. Firing Period Analysis of Uncoupled Linking PCNN

Set the initial value of feedback input *F*, dynamic threshold *E* to 0, and ignition state *Y* to 1; then the output neuron (pixel) firing state of each iteration is:

(1) When *n* = 0, which is the initial stage, $U_{ij}(0)=F_{ij}(0)=S_{ij}$, $E_{ij}(0)=0$, $Y_{ij}(0)=1$, neurons fire for the first time;
(2) When *n* = 1, $U_{ij}(1)=F_{ij}(1)=S_{ij}+e^{-a_{F}}S_{ij}$, $E_{ij}(1)=V_{E}$, $Y_{ij}(1)=0$, neurons extinguish;
(3) When *n* = 2, $U_{ij}(2)=F_{ij}(2)=S_{ij}+e^{-a_{F}}(S_{ij}+e^{-a_{F}}S_{ij})$, $E_{ij}(2)=e^{-a_{E}}V_{E}$, $Y_{ij}(2)=\varepsilon [S_{ij}+e^{-a_{F}}(S_{ij}+e^{-a_{F}}S_{ij})-e^{-a_{E}}V_{E}]$.

From the above, we can conclude that when *n* = 3…, $U_{ij}(n)=F_{ij}(n)=S_{ij}(1+e^{-a_{F}}+\cdots +e^{-na_{F}})$, $E_{ij}(n)=e^{-(n-1)a_{E}}V_{E}$, $Y_{ij}(n)=\varepsilon [S_{ij}(1+e^{-a_{F}}+\cdots +e^{-na_{F}})-e^{-(n-1)a_{E}}V_{E}]$.

Suppose neurons fire for the second time at *n* = *n*_1,_ then:

$$U_{ij}(n_{1})=F_{ij}(n_{1})=S_{ij}(1+e^{-a_{F}}+\cdots +e^{-n_{1}a_{F}})=\frac{S_{ij}(1-e^{-(n_{1}+1)a_{F}})}{1-e^{-a_{F}}},\;E_{ij}(n_{1})=e^{-(n_{1}-1)a_{E}}V_{E},Y_{ij}(n_{1})=1;$$

According to the condition for neuron fire $U_{ij}(n_{1})=E_{ij}(n_{1})$, we have $U_{ij}(n_{1})=E_{ij}(n_{1})\Rightarrow U_{ij}(n_{1})=\frac{S_{ij}(1-e^{-(n_{1}+1)a_{F}})}{1-e^{-a_{F}}}=e^{-(n_{1}-1)a_{E}}V_{E}\Rightarrow n_{1}=1+\frac{1}{a_{E}}\ln(\frac{V_{E}}{U_{ij}\left(n_{1}\right)})$. Let $k=\frac{1-e^{-(n_{1}+1)a_{F}}}{1-e^{-a_{F}}}$; therefore, $n_{1}=1+\frac{1}{a_{E}}\ln(\frac{V_{E}}{kS_{ij}})$.

In the $n=(n_{1}+1),(n_{1}+2),(n_{1}+3)\ldots$ moment, the value of the dynamic threshold $E_{ij}$ decays exponentially again, and the general formula becomes:

$$Y_{ij}(n)=0,\;U_{ij}(n)=S_{ij}(1+e^{-a_{F}}+\cdots +e^{-na_{F}}),\;E_{ij}(n)=(e^{-a_{E}}kS_{ij}+V_{E})e^{-(n-n_{1}-1)a_{E}}.$$

Suppose neurons fire for the third time at *n* = *n*_2_, then:

$$U_{ij}(n_{2})=F_{ij}(n_{2})=S_{ij}(1+e^{-a_{F}}+\cdots +e^{-n_{2}a_{F}})=\frac{S_{ij}(1-e^{-(n_{2}+1)a_{F}})}{1-e^{-a_{F}}},\;E_{ij}(n_{2})=e^{-(n_{2}-n_{1}-1)a_{E}}(V_{E}+e^{-a_{E}}kS_{ij}),\;Y_{ij}(n_{2})=1;$$

From the conditions for neuron fire, we have:

$$U_{ij}(n_{2})=E_{ij}(n_{2})\Rightarrow U_{ij}(n_{2})=\frac{S_{ij}(1-e^{-(n_{2}+1)a_{F}})}{1-e^{-a_{F}}}=e^{-(n_{2}-n_{1}-1)a_{E}}(V_{E}+e^{-a_{E}}kS_{ij})\Rightarrow n_{2}=1+n_{1}+\frac{1}{a_{E}}\ln(\frac{e^{-a_{E}}kS_{ij}+V_{E}}{U_{ij}\left(n_{2}\right)}),\;\mathrm{let}\;k'=\frac{1-e^{-(n_{2}+1)a_{F}}}{1-e^{-a_{F}}};\;\mathrm{therefore},\;n_{2}=1+n_{1}+\frac{1}{a_{E}}\ln(\frac{e^{-a_{E}}kS_{ij}+V_{E}}{k^{'}S_{ij}})$$

Suppose neurons fire for the fourth time at *n* = *n*_3_, then:

$$U_{ij}(n_{3})=E_{ij}(n_{3})\Rightarrow n_{3}=1+n_{2}+\frac{1}{a_{E}}\ln(\frac{e^{-a_{E}}k'S_{ij}+V_{E}}{U_{ij}\left(n_{3}\right)}),\;\mathrm{let}\;k''=\frac{1-e^{-(n_{3}+1)a_{F}}}{1-e^{-a_{F}}},\;\mathrm{therefore}\;n_{3}=1+n_{2}+\frac{1}{a_{E}}\ln(\frac{e^{-a_{E}}k'S_{ij}+V_{E}}{k''S_{ij}})$$

From the above, we can conclude that when *n*_4_…, $n_{m}=1+n_{m-1}+\frac{1}{a_{E}}\ln(\frac{e^{-a_{E}}cS_{ij}+V_{E}}{c'S_{ij}}),\begin{array}{cc} & m=4,5,\ldots ,N \end{array}$. where $c=\frac{1-e^{-(n_{m-1}+1)a_{F}}}{1-e^{-a_{F}}}$, $c'=\frac{1-e^{-(n_{m}+1)a_{F}}}{1-e^{-a_{F}}}$.

From the above analysis, we can conclude that the firing period $T_{ij}$ for uncoupled linking PCNN is:

(5) $$T_{ij}=n_{m}-n_{m-1}=1+\frac{1}{a_{E}}\ln(\frac{e^{-a_{E}}cS_{ij}+V_{E}}{c'S_{ij}})$$

### 2.2. Uncoupled Linking Chaotic PCNN

**Figure 2:**
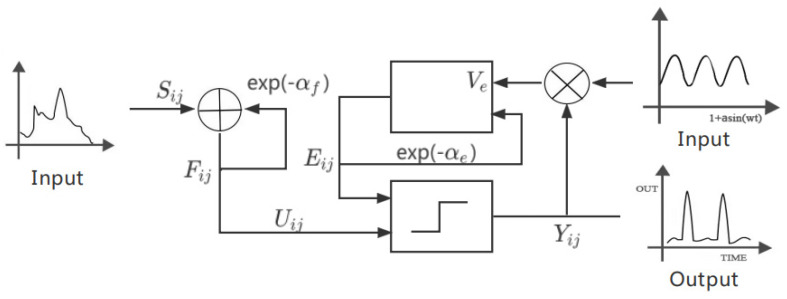
Simplified model of uncoupled linking chaotic PCNN.

It can be inferred from Section 2.1.1 that when the initial value of the dynamic threshold of the uncoupled linking PCNN model is constant, the neuron’s pulse firing is periodic, and its firing period is shown in Equation (5). In order to obtain the discrete chaotic PCNN model, we changed the initial dynamic threshold value of the original PCNN model into the oscillatory reset voltage, and its simplified model is shown in Figure 2.

Substituting the oscillatory reset voltage $V_{E}(n)=V_{Em}(1+a_{0}\sin(wn))$ into Equation (5) to obtain Equation (6) below [16]:

(6) $$n_{m}-n_{m-1}=1+\frac{1}{a_{E}}\ln(\frac{e^{-a_{E}}cS_{ij}+V_{Em}(1+a_{0}\sin(wn(m-1)))}{c'S_{ij}})$$

where w is the angular frequency, and n is the neuron firing moment.

Multiply Equation (6) with w, and let x(m)=wn(m); then, we have:

(7) $$x_{m}=x_{m-1}+w+\frac{w}{a_{E}}\ln(\frac{e^{-a_{E}}cS_{ij}+V_{Em}(1+a_{0}\sin(x_{m-1}))}{c'S_{ij}})\begin{array}{cc} & \left(\mathrm{mod}2\pi \right) \end{array}$$

Equation (7) is the one-dimensional circular map of the uncoupled linking chaotic PCNN.

#### 2.2.1. Basic Bifurcation Behavior Analysis of Chaotic PCNN

According to the above analysis, the discrete chaotic map mathematical model used for image encryption in this paper is:

(8) $$x_{n+1}=x_{n}+w+\frac{w}{a_{E}}\ln(\frac{e^{-a_{E}}cS_{ij}+V_{Em}(1+a\sin(x_{n}))}{c^{\prime }S_{ij}})\begin{array}{cc} & \left(\mathrm{mod}2\pi \right) \end{array}$$

where $c=c^{\prime }=\frac{1-e^{-(n+1)a_{F}}}{1-e^{-a_{F}}}$, $a_{F}$, $V_{Em}$, $a_{E}$, and $S_{ij}$ are controlling parameters, with set fixed parameters n=500, a=0.8, and w=7.7.

**Figure 3:**
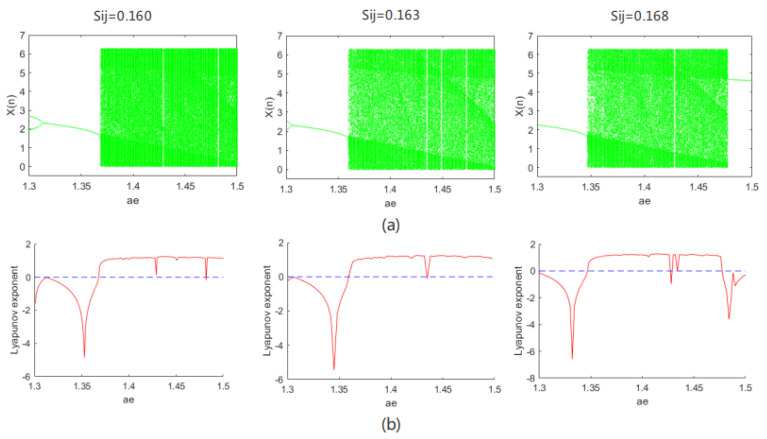
(**a**) Bifurcation; (**b**) Lyapunov exponent.

**Figure 4:**
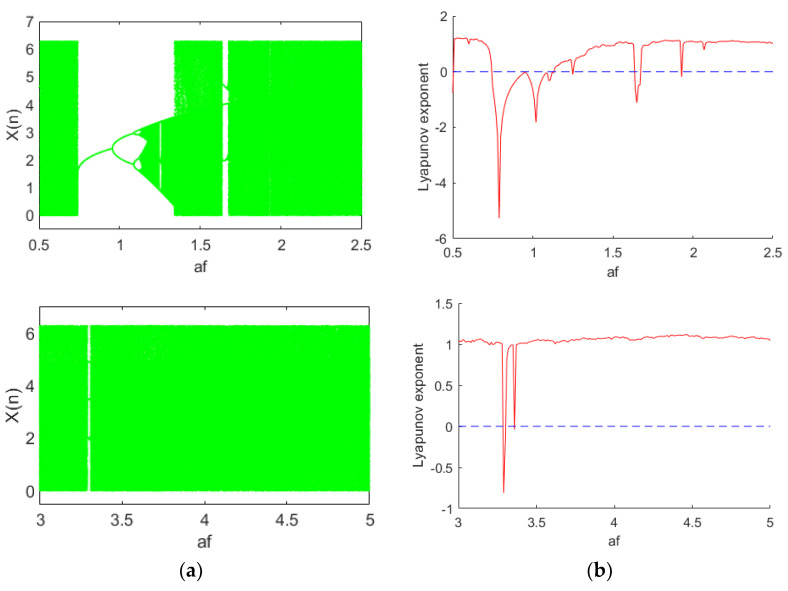
(**a**) Bifurcation; (**b**) Lyapunov exponent.

When setting initial value $x_{{}_{0}}=3$, the controlling parameters are $a_{F}=0.12$, $V_{Em}=17.2$, and $S_{ij}=0.16$. When parameter $a_{E}$ changes between [1.3, 1.5], the bifurcation diagram and Lyapunov exponent spectrum with respect to x are shown in Figure 3a,b, respectively. We know from Figure 3 that, as parameter $a_{E}$ changes, the system has different dynamic oscillation behaviors such as period, multiple periods, chaos, etc. When $a_{E}$ is within [1.3, 1.368], the system has a periodic solution and is in a periodic motion state when the maximum Lyapunov exponent is less than zero; when $a_{E}$ is within [1.369, 1.481], the system has chaotic dynamic behavior, where the maximum Lyapunov exponent is greater than zero; when fixing initial value $x_{{}_{0}}=3$, controlling parameters $S_{ij}=0.16$, $V_{Em}=17.2$, and $a_{E}=1.5$, as in Figure 4, when $a_{F}$ is within [1.631, 1.674], the system has a periodic solution and is in a periodic motion state; when $a_{F}$ is within [2.072, 2.5], the system has chaotic dynamic behavior. Based on the same reasoning, the bifurcation diagram and Lyapunov exponent spectrum of the system as a function of other parameters can also be obtained.

**Figure 5:**
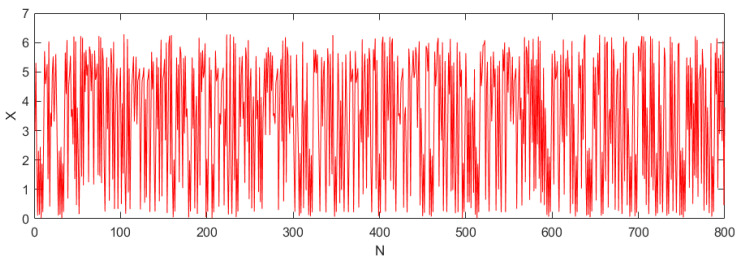
Chaos diagram of the system.

From the definition of Lyapunov exponent, when the Lyapunov exponent is greater than zero, the system is in a chaotic state. When fixing $x_{{}_{0}}=3$, with controlling parameters $a_{F}=0.12$, $V_{Em}=17.2$, and $S_{ij}=0.16$, from Figure 3b, when $a_{E}=1.5$, the Lyapunov exponent is greater than zero, and the system is in a chaotic state; its chaos diagram is shown in Figure 5.

#### 2.2.2. Non-Periodicity Analysis of Sequence

**Figure 6:**
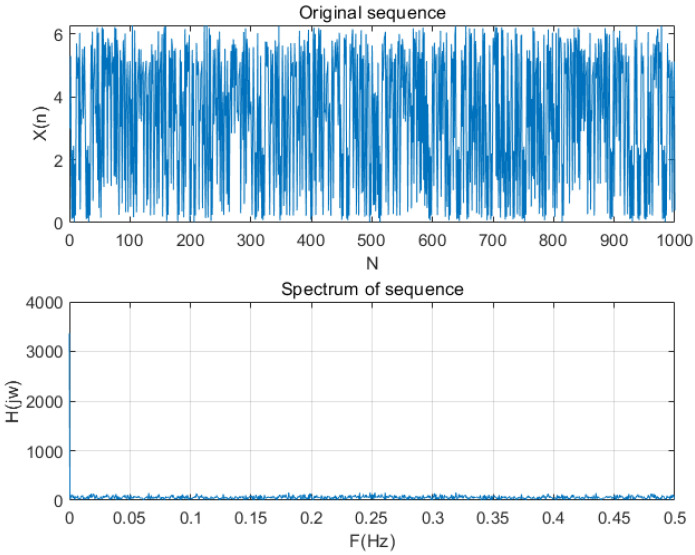
The sequence generated by chaotic system and its corresponding spectrum.

**Figure 7:**
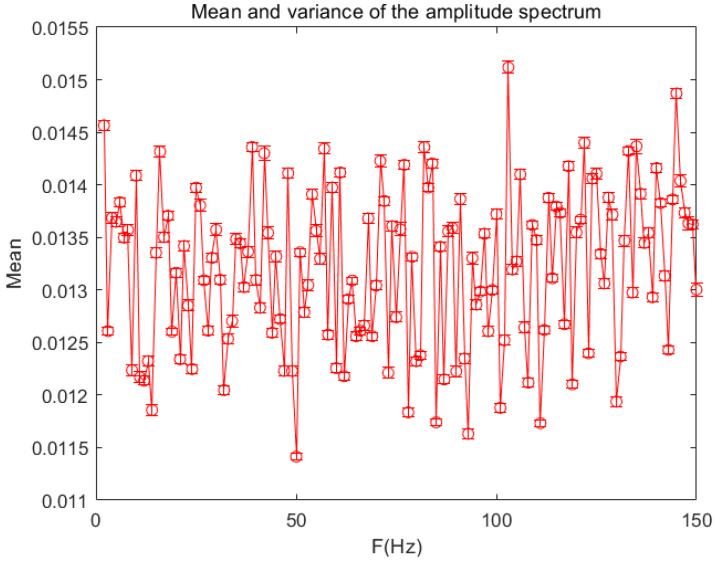
The mean variance diagram of amplitude spectrum (partial samples).

In order to verify the non-periodicity of the sequence generated by the chaotic pulse-coupled neural network proposed in this paper, we used the Fourier transform to conduct a lot of experiments and analyses. The magnitude spectrum is shown in Figure 6, and the mean variance diagram of the magnitude spectrum is shown in Figure 7. The analysis results show that the spectrum has no peak in the frequency domain. Since the random sequence generated by our algorithm have the same spectrum characteristics, the generated sequence is a non-periodic sequence.

### 2.3. Improved Arnold Transform Image Scrambling Method

The traditional Arnold transformation mainly uses Equation (9) to transform the pixels in the image from position (*x*, *y*) to position (*x*_1_, *y*_1_) and traverses all the pixels of the image through Arnold transformation so that the pixels of the entire image are scrambled to achieve the effect of image encryption [17].

(9) $$\left(\begin{array}{l} x_{1}\\ y_{1} \end{array}\right)=\left(\begin{array}{l} \begin{array}{cc} a & b \end{array}\\ \begin{array}{cc} c & d \end{array} \end{array}\right)\left(\begin{array}{l} x\\ y \end{array}\right)\mathrm{mod}N$$

where *a*, *b*, *c*, and *d* are parameters that satisfy *ad* − *bc* = 1, and *N* is the order of the image matrix.

**Figure 8:**
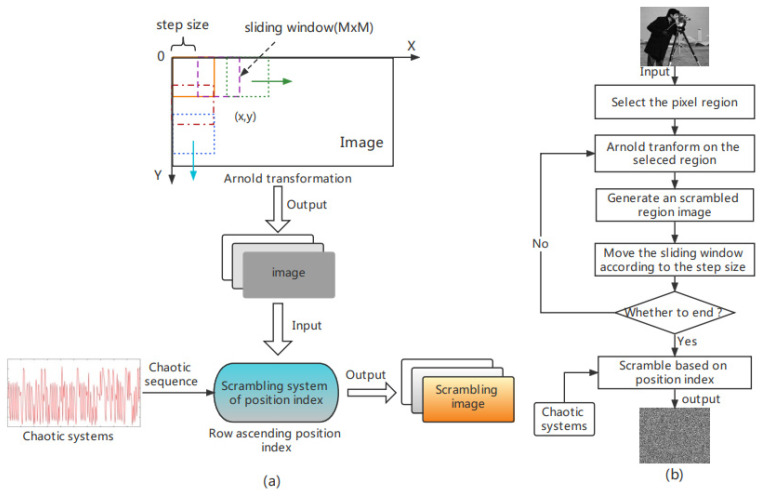
The (**a**) structure diagram and (**b**) flow chart of improved Arnold transform image scrambling method.

For the traditional Arnold transform, although it can scramble the position of image pixels simply and efficiently, it has the following shortcomings: (1) only square images can be scrambled, which reduces the scope of practical applications [18]; (2) it has periodicity, is easy to be deciphered by illegal persons, and the security is poor; as shown in Table 1, the period of Arnold transformation is related to the image size [19]. Illegal persons can reconstruct the original image according to the periodicity of Arnold transformation, which is not conducive to the security of image encryption; and (3) the key space is small, and the anti-attack ability is poor. In order to solve the shortcomings of the traditional Arnold transform and further improve the security of digital image encryption, this paper improves the Arnold transform. First, in order to solve the problem that the traditional Arnold transform can only scramble the square image, this paper uses a square sliding window (M × M) of variable size to traverse the image from left to right and from top to bottom. During the sliding process of the window, the pixels in the window undergo Arnold transformation, and the step size of the sliding window can be set according to actual needs. Secondly, due to the periodicity of the Arnold transform, for cryptography, the periodic encryption method is easy to be illegally cracked, and the security is not high. Therefore, this paper uses the proposed CPCNN chaos system to generate the chaos sequence and sort the chaotic sequence to obtain the image position index value. According to the position index value, the image after Arnold transformation is scrambled with a special level to make it lose the original periodicity of the Arnold transformation. At the same time, the method has a large key space, which can solve the problems of small key space and poor security of Arnold transform. The structure diagram and flow chart of this method are shown in Figure 8.

## 3. Scheme and Process of Image Encryption and Decryption

The image encryption algorithm proposed in this paper is mainly divided into two parts: (1) pixel replacement, using the chaotic pulse-coupling neural network to generate a chaotic sequence, taking the modulo of the chaotic sequence, and performing XOR operation with the original image to obtain a pre-encrypted image and (2) pixel scrambling, using the improved Arnold transform in this paper to scramble the pixel position of the pre-encrypted image to obtain the final encrypted image.

**Figure 9:**
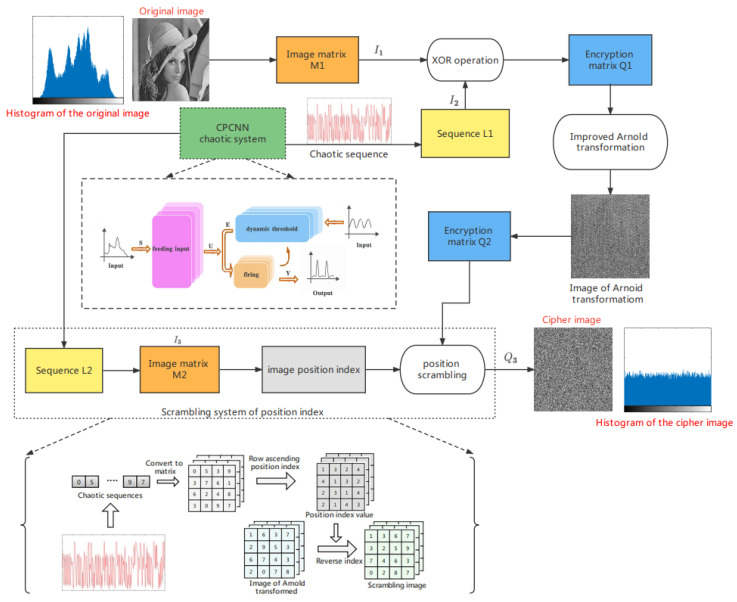
Flow chart of image encryption method.

The flow chart of encryption in this paper is shown in Figure 9. Algorithm 1 gives the steps involved in the encryption process. The specific encryption steps are as follows:

Step 1: Image input, parameter initialization. Input the grayscale image of size W × H, and convert it into a one-dimensional matrix *I*_1_; set the initial parameter value of the chaotic system. See Section 4.2 for the initial parameter value;

Step 2: Generate chaotic sequence *L*1. Use discrete CPCNN to iterate M × N times to generate a chaotic sequence of length M × N;

Step 3: Normalize the sequence *L*1. The sequence generated by the chaotic system is normalized by Equation (10), and the sequence is converted into the range of [0, 1] to obtain the sequence *L*3;

(10) $$L3\left(i\right)=\frac{L1\left(i\right)-\max(L1)}{\max(L1)-\min(L1)}$$

where max/min refers to the maximum/minimum value of the sequence obtained.

Step 4: Generate an encrypted key sequence. The key sequence *I*_2_ is obtained by converting the sequence *L*3 into an integer in the range of [0, 255] using the modulo operation of Equation (11), where “sum” in Equation (11) refers to the sum of all pixel values of the input grayscale image;

(11) $$\begin{array}{c} I_{2}=floor(\mathrm{mod}((L3\cdot sum)\cdot 10^{8},256))\\ I_{2}=floor(\mathrm{mod}((L3)\cdot 10^{8},256)) \end{array}\}\begin{array}{c} sum\neq 0\\ sum=0 \end{array}$$

where mod in Equation (11) is the modulo function, and *floor* is the rounding function.

Step 5: Preliminary encryption operation. XOR the one-dimensional matrix *I*_1_ with the key sequence *I*_2_ to generate a new random sequence, as shown in Equation (12), and then convert the new random sequence into a pre-encrypted image *Q*1 of size W × H;

(12) $$Q1=bitxor(I_{1},I_{2})$$

where bitxor in Equation (12) refers to the bitwise exclusive OR operation.

**Figure 10:**
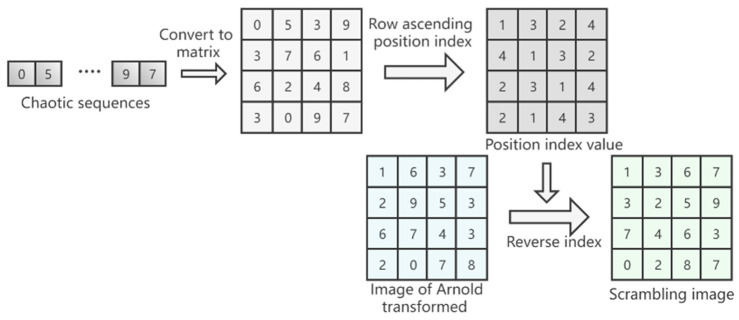
Flow chart of steps 7–9.

Step 6: Arnold transform on the pre-encrypted image. Using a 100 × 100 square sliding window and setting the step size to 30, the pre-encrypted image is traversed, and the entire pre-encrypted image is Arnold transformed to obtain a new scrambled image *Q*2;

Step 7: Repeat the process of steps 2–4 to generate a new key sequence I_3_, and convert the key sequence I_3_ into an image matrix M2 with a size of W × H;

Step 8: Row the image matrix M2 in ascending order and obtain its position index value at the same time. If the pixel value of the first row of image matrix M2 is assumed to be [0,5,3,9], the position index value obtained in ascending row order is [1,3,2,4];

Step 9: Use the image position index value generated in step 8 to reverse the index the scrambled image *Q*2 generated in step 6 to obtain the final encrypted image *Q*3. If the pixel value of the first row of the image *Q*2 is [1,6,3,7], use the position index value [1,3,2,4] of step 8 to perform reverse indexing to obtain the pixel of the first row of the final encrypted image *Q*3; the value is [1,3,6,7]. The flowchart of steps 7–9 is shown in Figure 10.

The decryption process of the encryption algorithm proposed in this paper is the inverse process of the encryption process. As long as the encryption process is reversed, the original plaintext image can be restored without distortion. This paper will not describe it in detail.
**Algorithm 1:** Proposed decryption algorithm.**Input:** Image M1 of size W × H. **Output:** Encryption result *Q*3. 1: Set the initial parameter value of the chaotic system, set fixed parameters n=500, a=0.8, w=7.7; controlling parameters $a_{F}=0.12$, $V_{Em}=17.2$, $S_{ij}=0.16$, $a_{E}=1.5$; initial value $x_{{}_{0}}=3$;2: Generate chaotic sequence L_1_ and L_2_ using proposed CPCNN map; 3: sum = sum(M1); 4: $I_{1}$ = reshape (M1, 1, W × H);5:  **for** *i* = 1 to L1 **do** 6:   L3(i)=(L1(i)−max(L1))/(max(L1)−min(L1)); 7:   **if** *sum* ≠ 0 **then** 8:   $I_{2}=floor((L3\cdot sum\cdot 10^{8})\mathrm{mod}256)$; 9:   **else** 10:   $I_{2}=floor((L3\cdot 10^{8})\mathrm{mod}256)$; 11:   **end if** 12:  **end for** 13: Q2 = reshape ($I_{1}⊕I_{2}$, W, H); 14: Using a 100 × 100 square sliding window and setting the step size to 30, the pre-encrypted image is traversed, and the entire pre-encrypted image is Arnold transformed to obtain a new scrambled image *Q*2; 15: M2 = reshape (L2, W, H); 16: index = zeros (W, H); 17: **for** *i* = 1 to W **do** 18:  [~, index (*i*, :)] =sort (M2 (*i*, :)); 19: **end for**20: count = W; 21: **for** *i* = 1 to W **do** 22:  **for** *j* = 1 to H **do** 23:   *Q*3 (*i*, *j*) = *Q*2 (index (*i*, *j*), count); 24:  **end for**
25:  count = count − 1;26: **end for**

## 4. Experiment Environment and Results

### 4.1. Experiment Environment

The equipment environment used in this experiment was Windows 10 (64-bit) operating system with 16 G memory; the processor of the running platform was Intel (R) Core (TM) i7-10510U CPU @ 1.80 GHz 2.30 GHz; GPU is NVIDIA GeForce MX250; Development and testing software environment was MATLAB R2019a.

### 4.2. Experiment Parameter Setting and Result

Parameter setting: for the experiment in this paper, set fixed parameters for CPCNN mapping n=500, a=0.8, w=7.7; controlling parameters $a_{F}=0.12$, $V_{Em}=17.2$, $S_{ij}=0.16$, $a_{E}=1.5$; and initial value $x_{{}_{0}}=3$. Set the Arnold transform parameters as a=1, b=1,c=2, and d=3.

Sliding window parameter setting: The choice of the window size will affect the speed of encryption and the effect of encryption. Therefore, in order to weigh the efficiency of encryption and the effect of encryption, Formulas (13) and (14) are recommended to determine the window size (*N* × *N*) and step size (S), respectively.

(13) $$\frac{W}{3}\leq N\leq \frac{2\times W}{12}\begin{array}{cc} & \left(W\leq L\right) \end{array}$$

(14) $$\frac{N}{4}\leq S\leq \frac{N}{3}$$

In the above formula, *L* and *W* are the length and width of the input image, *N* is the size of the sliding window, and *S* is the sliding step. *N* and *S* must be integers.

**Figure 11:**
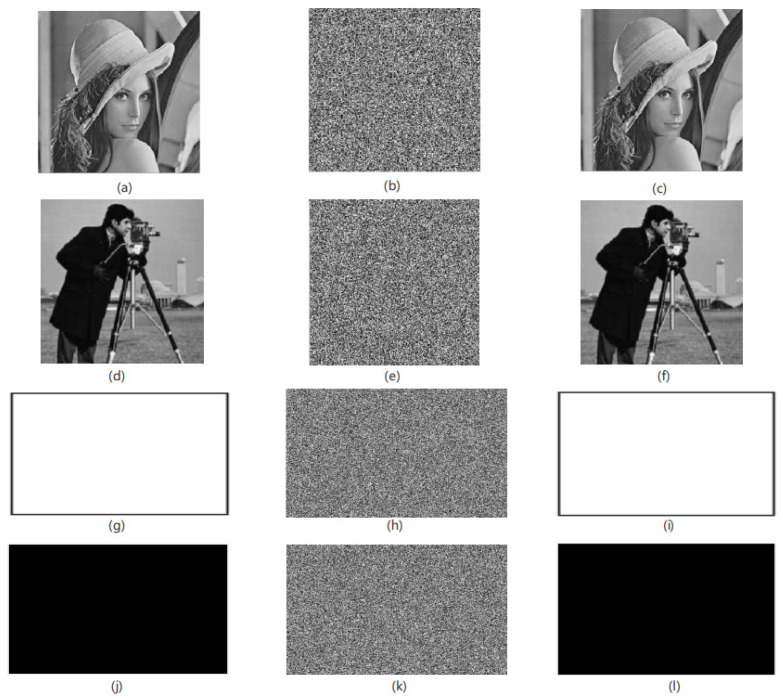
The encryption and decryption effect of the algorithm in this paper. (**a**) Plain-text image of Lena image (original image), (**b**) encrypted image, (**c**) decrypted image, (**d**) plain-text image of Cameraman image, (**e**) encrypted image, (**f**) decrypted image, (**g**) plain-text image of White image, (**h**) encrypted image, (**i**) decrypted image, (**j**) plain-text image of Black image, (**k**) encrypted image, (**l**) decrypted image.

Result: The algorithm proposed in this paper can implement encryption processing for images of any size. In the experimental simulation, this paper uses standard Lena, Cameraman, White and Black grayscale images as encrypted images, and the image sizes are 256 × 256 and 300 × 400. As shown in Figure 11, Figure 11a,d,g,j are the encrypted images of Figure 11b,e,h,k obtained after the original images are encrypted by the algorithm in this paper. Comparing the images before and after encryption, it can be seen that, after the original images are encrypted, the original feature information of the images is completely lost, and the algorithm in this paper has a good encryption effect. At the same time, after the encrypted image is decrypted, the information characteristics of the original images can be restored accurately and losslessly, as shown in Figure 11c,f,i,l.

## 5. Security Analysis

### 5.1. Statistical Characteristic Analysis of Ciphertext

#### 5.1.1. Histogram Statistical Analysis

**Figure 12:**
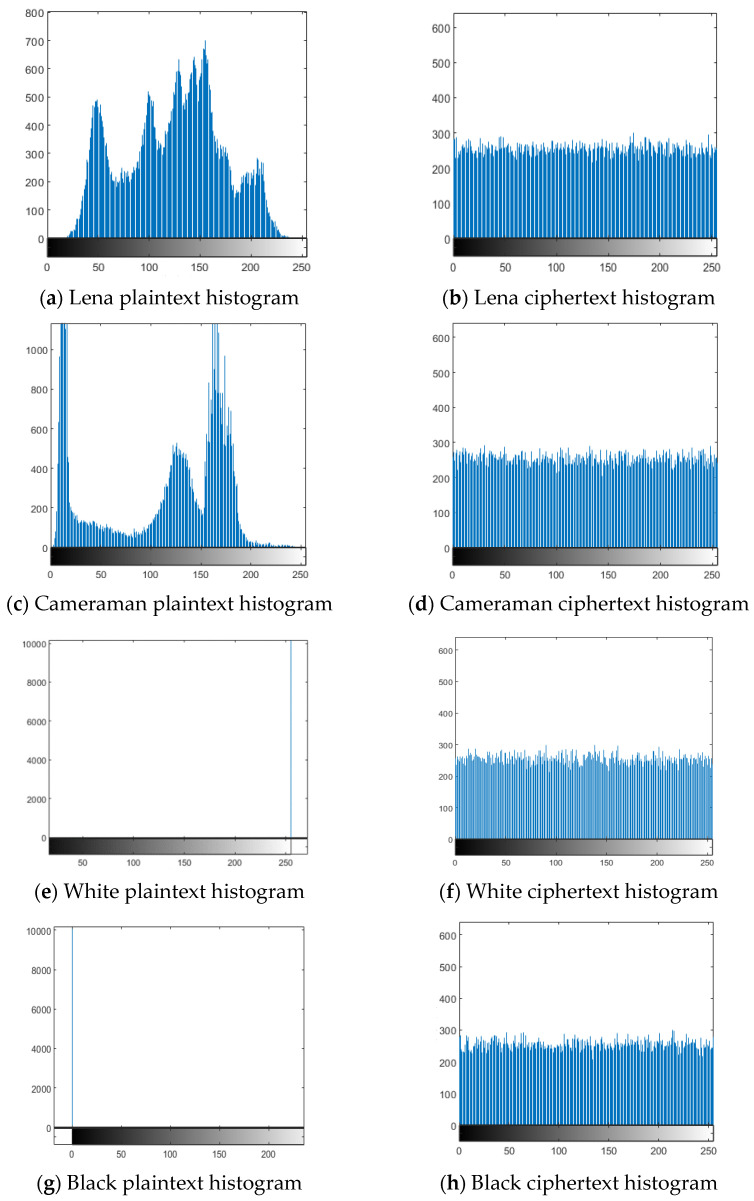
Image pixel distribution histogram.

The grayscale histogram of the image can intuitively reflect the distribution and distribution rules of each grayscale value. Statistical analysis attack means that the illegal persons can crack the encrypted image by comparing and analyzing the image statistical law and image gray value distribution information of the plaintext and ciphertext of the image [20]. Therefore, the grayscale histogram of the image can reflect the ability of the algorithm in this paper to resist statistical analysis attacks to a certain extent. The more uniform the distribution of the gray histogram of the image, the smaller the corresponding variance. The smaller the variance, the less statistical information contained in the image, the stronger the ability to resist statistical analysis attacks and the higher the security. According to the histogram of pixel distribution before and after encryption in Figure 12, it can be seen that the histogram of the plaintext image is unevenly distributed and has rich image statistical information. After image encryption processing, the histogram distribution of the image is relatively uniform, indicating that the encryption algorithm in this paper breaks the statistical feature information of the original image and can effectively resist the statistical analysis attack of illegal persons.

In order to quantitatively analyze the uniformity of the histogram distribution, this paper uses the histogram $\chi^{2}$ distribution to verify. Equation (15) is the equation to calculate histogram distribution, where $H_{i}$ is the number of image pixel values *i*, and the value range *i* is [0, 255].

(15) $$\chi^{2}=\frac{1}{256}\sum_{i=0}^{255}{\left(H_{i}-\frac{1}{256}\sum_{i=0}^{255}H_{i}\right)}^{2}$$

In this paper, the significance level a=0.05 is used for verification, and Equation (15) is used to calculate the original and encrypted images of Lena, Cameraman, White, and Black, and the obtained values are shown in Table 2. The values of the ciphertext images are 237.8047, 257.2188, 263.6016, and 260.2813 and are less than $\chi_{0.05}^{2}\left(255\right)=293.24783$. Therefore, the histogram of the ciphertext image can be considered to meet the uniform distribution at the significance level a=0.05, and it also shows that the algorithm in this paper can change the histogram distribution of the original image well and has a good ability to resist statistical analysis attacks.

#### 5.1.2. Correlation Analysis of Adjacent Pixels

**Figure 13:**
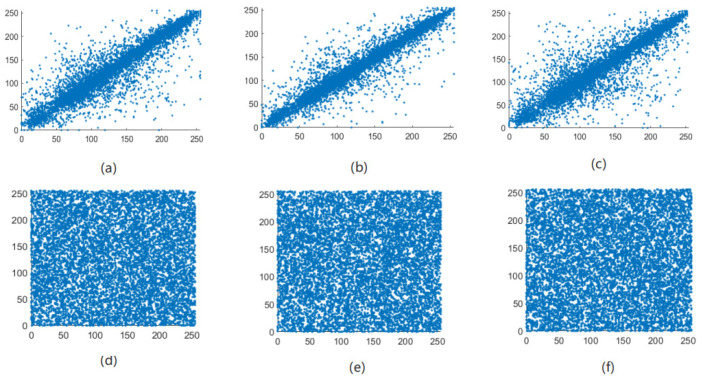
Lena scatter distribution map of adjacent pixels before and after image encryption. (**a**) Horizontal, (**b**) vertical, and (**c**) diagonal directions of the plain Lena image and (**d**) horizontal, (**e**) vertical, and (**f**) diagonal directions of the encrypted Lena image.

**Figure 14:**
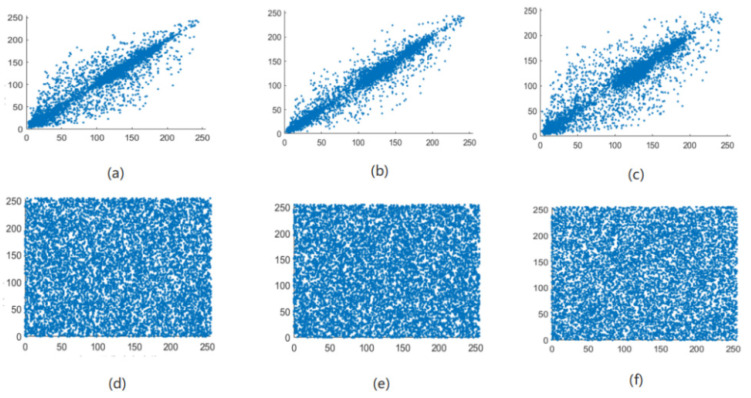
Cameraman scatter distribution map of adjacent pixels before and after image encryption. (**a**) Horizontal, (**b**) vertical, and (**c**) diagonal directions of the plain Cameraman image and (**d**) horizontal, (**e**) vertical, and (**f**) diagonal directions of the encrypted Cameraman image.

**Figure 15:**
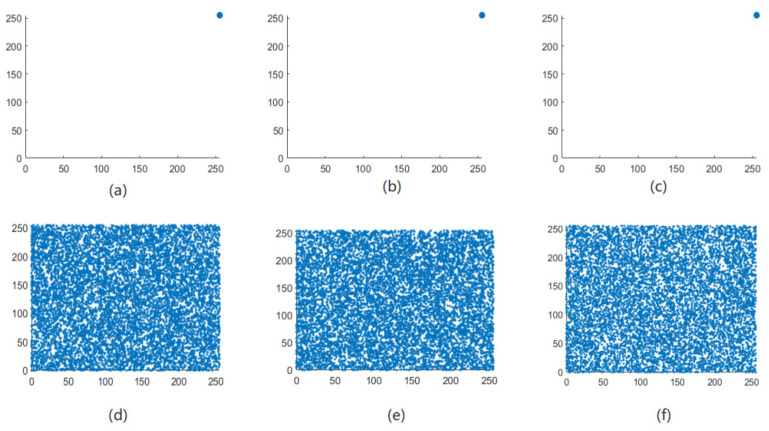
White scatter distribution map of adjacent pixels before and after image encryption. (**a**) Horizontal, (**b**) vertical, and (**c**) diagonal directions of the plain White image and (**d**) horizontal, (**e**) vertical, and (**f**) diagonal directions of the encrypted White image.

**Figure 16:**
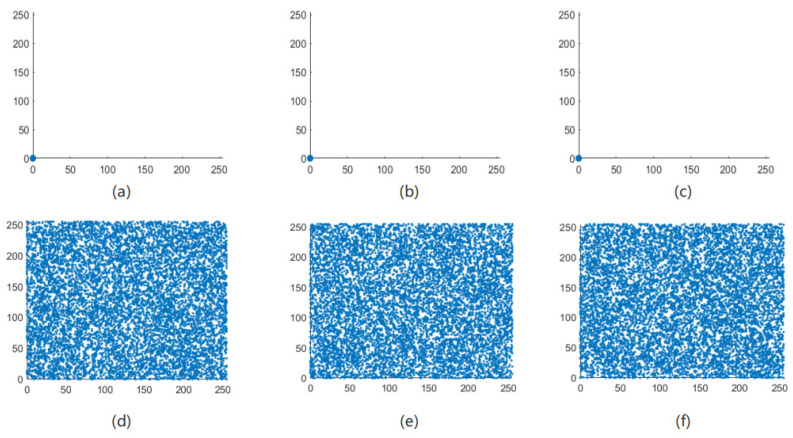
Black scatter distribution map of adjacent pixels before and after image encryption. (**a**) Horizontal, (**b**) vertical, and (**c**) diagonal directions of the plain Black image and (**d**) horizontal, (**e**) vertical, and (**f**) diagonal directions of the encrypted Black image.

The correlation of correlation between adjacent pixels in an image reflects the degree of correlation and similarity between adjacent pixels. The closer the pixel values between adjacent pixels are, the stronger the correlation between adjacent pixels of the image, and the easier it is for illegal attackers to use the feature of strong correlation between adjacent pixels to infer the pixel value of surrounding pixels through a single pixel point [21]. The meaning of encryption is to break the original strong correlation between image pixels and reduce the correlation between adjacent pixels of the image. Therefore, the encryption effect of the algorithm in this paper can be judged by the correlation between the adjacent pixels of the image before and after encryption. As can be seen from Figure 13, Figure 14, Figure 15 and Figure 16, the adjacent pixel scatter plots of the plaintext image show a linear relationship, while the ciphertext image shows a uniform distribution.

In order to quantitatively analyze the correlation of adjacent pixels, this paper introduces the correlation coefficient to calculate the correlation strength in the horizontal, vertical, and diagonal directions of the image. The calculation formula of the correlation coefficient of adjacent pixels in the image is shown in Equation (16). In this paper, 10,000 adjacent pixels are randomly selected from the plaintext and ciphertext of the image for calculation. The calculation results are shown in Table 3.

(16) $$\begin{array}{c} \begin{array}{l} R_{\mathrm{xy}}=\frac{\mathrm{cov}(x,y)}{\sqrt{D(x)}\sqrt{D(y)}} \end{array}\\ \begin{array}{c} E(x)=\frac{1}{n}\sum_{i=1}^{n}x_{i}\\ D(x)=\frac{1}{n}\sum_{i=1}^{n}{\left(x_{i}-E(x)\right)}^{2}\\ \mathrm{cov}(x,y)=\frac{1}{n}\sum_{i=1}^{N}\left(x_{i}-E(x))(y_{i}-E(y)\right) \end{array} \end{array}\}$$

Among them, in Equation (16), *y* is the adjacent pixel of *x*, and *n* is the total number of image pixels; E(x) and D(x) are the mean and variance of the image pixel value; cov(x,y) is the covariance of two adjacent pixels *x* and *y*; and $R_{\mathrm{xy}}$ is the correlation coefficient of adjacent pixels. When the correlation coefficient is closer to 1, it indicates that the correlation of adjacent pixels is higher, and the closer it is to 0, the lower the correlation. It can be seen from Table 3 that the correlation coefficient of the plaintext image is close to 1, while the correlation coefficient of the ciphertext image is close to 0. Therefore, the encryption algorithm in this paper can well break the correlation between adjacent pixels of the original image to achieve a good encryption effect.

### 5.2. Information Entropy Analysis

Information entropy is an important indicator used to measure the randomness of the signal source, and its calculation formula is as Equation (17). This paper uses information entropy to quantitatively compare the randomness of images before and after encryption. The larger the information entropy value, the stronger the randomness of the information distribution of the image and the better the encryption effect [22]. The ideal information entropy of the ciphertext image is eight, indicating that the information distribution of the image is completely random. It can be easily obtained from Table 4 that the information entropy of the encrypted images is greater than 7.997. Therefore, it can be shown that the encryption effect of this paper is better, and the security of the encrypted image is higher.

(17) $$H(x)=-\sum_{i=0}^{M-1}P(x_{i})\log_{2}P(x_{i})$$

where *M* = 256 in Equation (17) represents all states of image pixel values (0–255), $x_{i}$ represents the pixel value of the image, and $P(x_{i})$ refers to the probability that the number of pixels with corresponding pixel value $x_{i}$ accounts for the total number of the entire image.

### 5.3. Tests for Randomness

In order to test the randomness of the generated sequences, TestU01 test was used to verify the statistical characteristics of the proposed system. For each test, a *p*-value was calculated. If the *p*-value was in the range [10^−4^, 1−10^−4^], the test was successful [23]. Any *p*-value outside this range is considered a failed test. Table 5 shows the final test results of TestU01; it can be seen that the sequences produced by the chaotic system proposed in this paper can pass the TestU01 test. The experimental results show that the chaotic sequence generated by chaotic pulse-coupled neural network has good randomness and is safe and reliable.

At the same time, in order to further verify the randomness, we also introduced the performance index of the average neighborhood gray difference for quantitative analysis and comparison. The degree of gray difference is another statistical measure to compare the randomness between the original image and the encrypted image [24]. The final result of the Equations (18)–(20) is called the GVD score. The GVD score is closer to 0 if the two images are closer, and closer to 1 otherwise. The GVD results of our algorithm are shown in Table 6. Since the GVD score for encrypted images obtained by our algorithm is closer to one, the randomness is quite good.

(18) $$GVD=\frac{AN^{'}[GN(x,y)]-AN[GN(x,y)]}{AN^{'}[GN(x,y)]+AN[GN(x,y)]}$$

(19) $$AN[GN(x,y)]=\frac{\sum_{x=2}^{M-1}\sum_{y=2}^{N-1}GN(x,y)}{\left(M-2)(N-2\right)}$$

(20) $$GN(x,y)=\frac{\sum {\left[G(x,y)-G(x^{'},y^{'})\right]}^{2}}{4}\begin{array}{cc} & here(x^{'},y^{'}) \end{array}=\{\begin{array}{c} \left(x-1,y\right)\\ \left(x+1,y\right)\\ \left(x,y+1\right)\\ \left(x,y-1\right) \end{array}$$

*G* (*x*, *y*) represents the gray value at position (*x*, *y*). *AN* and *AN*′ represent the average neighboring gray value before and after encryption, respectively.

### 5.4. Key Space Analysis

The key space refers to the set of all keys, that is, the value range of the keys. Illegal attackers usually use brute force attacks to decipher encrypted images by traversing all possible keys, so a large enough key space can effectively resist brute force attacks by illegal attackers [25]. The encryption algorithm in this paper uses parameters such as $a_{F}$, $V_{Em}$, $S_{ij}$, $a_{E}$, and $x_{{}_{0}}$ as encryption keys; if the value step size of each key is taken as 10^−16^, then $a_{F}$, $V_{Em}$, $S_{ij}$, $a_{E}$, and $x_{{}_{0}}$ have available key spaces of 10^12^, 10^12^, 10^13^, 10^14^, and 10^14^, respectively. From cryptographic theories, only a key space larger than 2^128^ can resist illegal brute force attacks. The key space of the encryption algorithm in this paper can reach above 10^130^, which is larger than 2^430^. Therefore, the encryption algorithm in this paper has a large enough key space and can effectively resist brute force attacks. The key space comparison results are shown in Table 7.

### 5.5. Key Sensitivity Analysis

**Figure 17:**
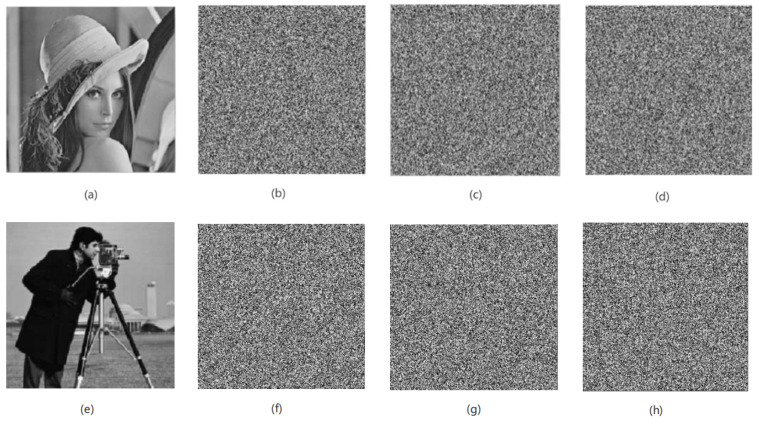
Decryption result after changing key. (**a**,**e**) Decryption image with correct key; (**b**,**f**) decrypted image with $V_{Em}{\;+\;10}^{-10}$; (**c**,**g**) decrypted image with $a_{E}{\;+\;10}^{-10}$; (**d**,**h**) decrypted image with $a_{F}{\;+\;10}^{-10}$.

An ideal encryption algorithm should have good key sensitivity; that is, making a small change to the key produces a completely different encryption result [31]. The stronger the key sensitivity of the encryption algorithm, the higher the security of encryption. In order to verify the key sensitivity of the algorithm in this paper, through qualitative analysis, only a small change is made to a single key value each time, and the difference between the two values before and after the change is only 10^−10^. It can be seen from Figure 17 that the correct plaintext image cannot be restored as long as the key is slightly changed in the decryption process, and the image obtained by making a slight change to a single key value is very different from the plaintext image. Therefore, it can be shown that the algorithm in this paper has a very good key sensitivity in the decryption process.

In order to further analyze the key sensitivity of the algorithm in the encryption process, this paper introduces two indicators, the Number of Pixel Change Rate (NPCR) and the Unified Average Change Intensity (UACI), to quantitatively evaluate the key sensitivity of the encryption process in this paper. NPCR and UACI, respectively, represent the proportion and average change intensity of the number of pixel changes between the two encrypted images, and the calculation formula is as Equation (21). The ideal NPCR and UACI values are 99.609375% and 33.463542%, respectively. The closer the NPCR and UACI values are to the ideal values, the higher the key sensitivity of the encryption algorithm and the higher the security. When the value of a single key increases by 10^−10^, the NPCR and UACI values are between the corresponding encrypted image and the original encrypted image, and the proportions of different pixels of the two encrypted images are shown in Table 8.

(21) $$\begin{array}{c} NPCR=\frac{\sum_{i,j}D(i,j)}{M\times N}\times 100\%\\ D(i,j)=\left\{ \begin{array}{l} 1,P_{1}(i,j)=P_{2}(i,j)\\ 0,P{\left(i,j\right)}_{1}\neq P_{2}(i,j) \end{array}\right.\\ UACI=\frac{1}{M\times N}\frac{\sum \left(P1(i,j)-P2(i,j)\right)}{255}\times 100\% \end{array}\}$$

where $P_{}(i,j)$ is the pixel at corresponding location (i,j) of the encrypted image, and *M* and *N* are the height and width of the image, respectively.

### 5.6. Anti-Attack Ability Analysis

#### 5.6.1. Anti-Differential Attack Analysis

**Figure 18:**
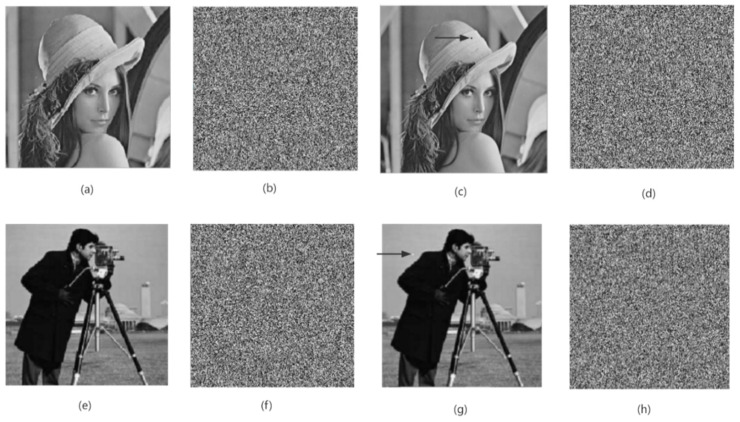
Comparison diagram before and after differential processing. (**a**,**e**) Plain-text image, (**b**,**f**) encrypted image, (**c**,**g**) plain-text image after differential processing, and (**d**,**h**) encrypted image after differential processing.

A differential attack means that an illegal attacker obtains a new encrypted image after making slight changes to the plaintext image, then compares it with the original encrypted image to find the data relationship and law between the two encrypted images, and then realizes the cracking of the ciphertext image [32]. As shown in Figure 18, this paper only changes the pixel value of one pixel of the original plaintext image (at the arrow in Figure 11c). By comparing the original ciphertext image and the encrypted image after changing a single pixel value, it is found that in the obtained two images, more than 99% of the pixels in the encrypted image are not identical, as shown in Table 9. At the same time, this paper also uses the NPCR and UACI values to make a quantitative comparison. As shown in Table 10, the calculation results of NPCR and UACI are closer to the ideal values. Therefore, it can be proved that the algorithm in this paper has a strong ability to resist differential attacks.

#### 5.6.2. Anti-Salt and Pepper, Gaussian Noise Attack Analysis

**Figure 19:**
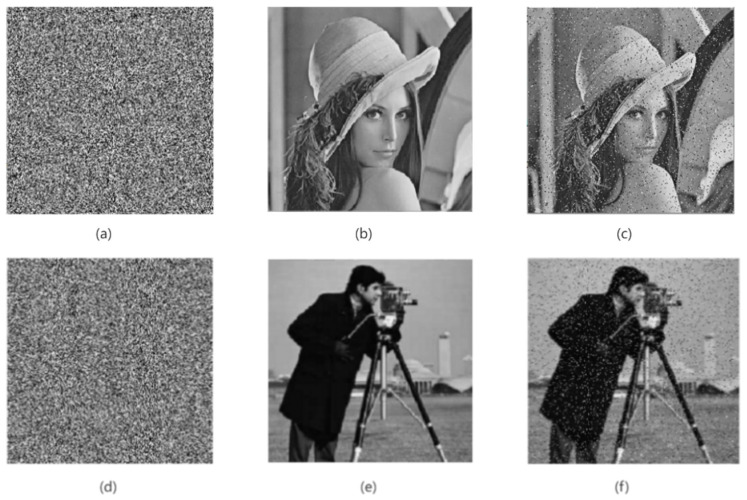
Ciphertext image with salt and pepper noise and its decryption graph. (**a**,**d**) Encrypted image with 0.1 salt and pepper noise; (**b**,**e**) plain-text image; (**c**,**f**) decrypted image.

**Figure 20:**
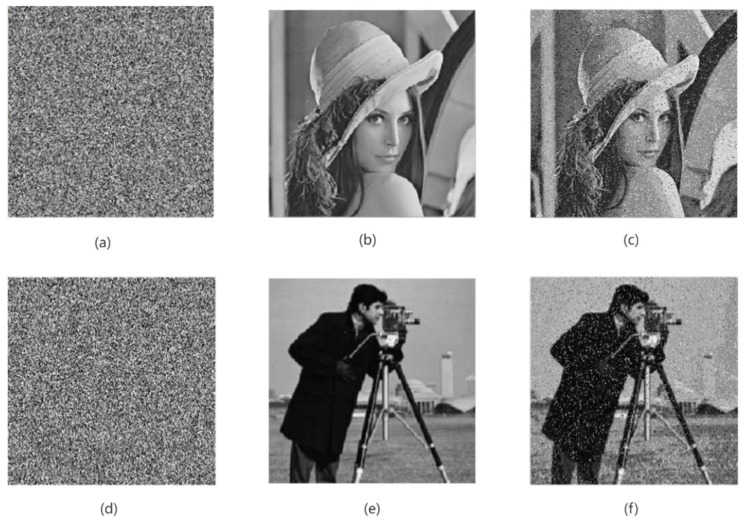
The ciphertext image with Gaussian noise and its decryption graph. (**a**,**d**) Encrypted image with Gaussian noise; (**b**,**e**) plain-text image; (**c**,**f**) decrypted image.

In the process of information transmission, the image will inevitably encounter the influence of various communication noises. When the encrypted image is attacked by noise, it will have a certain impact on the decryption of the image so that the ciphertext image is difficult to restore the clear original image [33]. Therefore, a good encryption algorithm should have the ability to resist noise attacks, and when the encrypted image is attacked by noise, it can restore the original image clearly. In order to verify the anti-noise ability of the encryption algorithm in this paper, this paper randomly adds 10% salt and pepper noise and Gaussian noise with a mean of 0 and a variance of 0.001 to the encrypted image, as shown in Figure 19 and Figure 20. It can be easily seen from Figure 19 and Figure 20 that after the noise processing of the encrypted image, we can still see the decrypted image information clearly. Therefore, the encryption algorithm in this paper has a good ability to resist noise attacks, and it also shows that the algorithm in this paper has strong robustness.

#### 5.6.3. Analysis of Anti-Shearing Attack Ability

**Figure 21:**
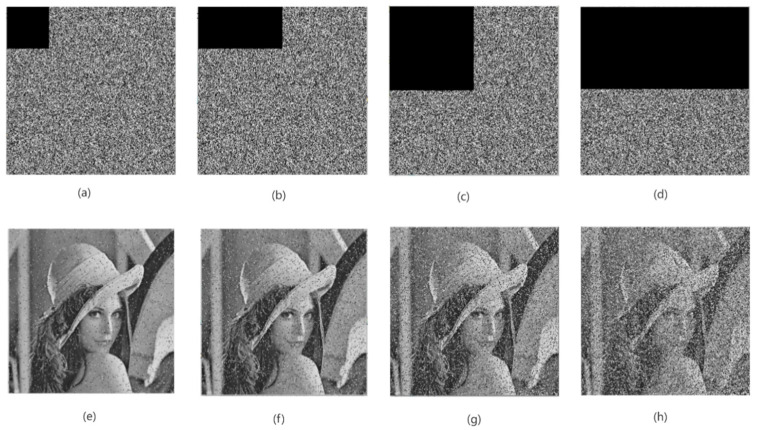

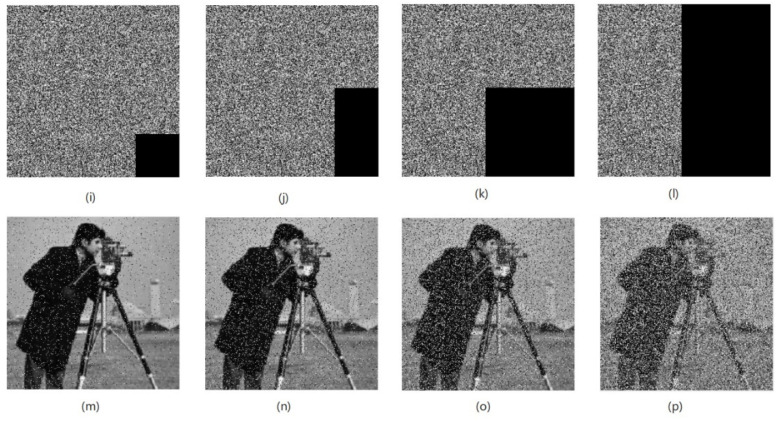
The cropped ciphertext image and its decryption renderings. Encrypted image with (**a**,**i**) 1/16 data loss, (**b**,**j**) 1/8 data loss, (**c**,**k**) 1/4 data loss, and (**d**,**l**) 1/2 data loss; decrypted image with (**e**,**m**) 1/16 data loss, (**f**,**n**) 1/8 data loss, (**g**,**o**) 1/4 data loss, and (**h**,**p**) 1/2 data loss.

Due to the fact that some data are easy to be lost or cropped during the image transmission process, a good decryption algorithm should have a good ability to resist cropping and data loss, even if some data are lost in the decryption process, basically restoring the original image [34]. In this paper, in order to verify the ability of the algorithm to resist cropping and data loss, the encrypted image is cropped by 1/16, 1/8, 1/4, and 1/2, and then the decrypted images obtained by decrypting there are shown in Figure 21. It can be seen from Figure 21 that even if the encrypted image loses some information, the characteristic information of the original image can be basically recovered after decryption, and the content in the image can be easily distinguished by the naked eye. Therefore, the algorithm in this paper has better ability to resist cropping and data loss.

#### 5.6.4. Noise Processing of Decrypted Images

**Figure 22:**
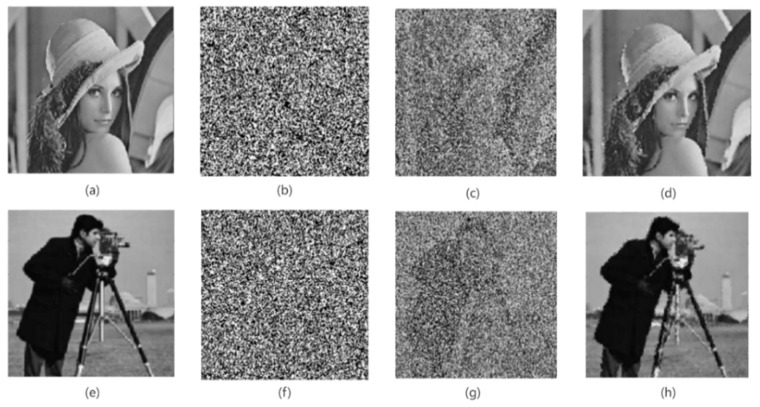
De-noising result. (**a**) Original image; (**b**) encrypted image with 0.7 salt and pepper noise; (**c**) decrypted image of (**b**); (**d**) denoising for decrypted image (**c**). (**e**) Original image; (**f**) encrypted image with 0.8 salt and pepper noise; (**g**) decrypted image of (**f**); (**h**) denoising for decrypted image (**g**).

Images are easily affected by various external factors during network transmission, resulting in decrypted images mixed with various noises. In order to filter out the noise in the image and restore the detailed information of the original image more clearly, this paper adopts the noise filtering method based on the multi-layer PCNN proposed by the author earlier. For the specific method, please refer to the literature [35]. This method mainly utilizes the pulse ignition feature of PCNN to locate the pepper noise and salt noise in the decrypted image. Then, without changing the size of the filter window, multi-layer PCNN is used to process the image to reduce the salt and pepper noise in the decrypted image and better restore the details of the original image. The experiments show that this method can filter out most of the salt and pepper noise in the decrypted image. The experimental results are shown in Figure 22. It can be seen from the figure that the image obtained after decryption has some salt and pepper noise, as shown in Figure 22c,g. After the decrypted image is processed by the noise filtering method of the multi-layer PCNN proposed by the author, the salt and pepper noise in the image can be effectively reduced, and the feature information of the original image can be better preserved.

### 5.7. Analysis of Speed

In addition to the evaluation of the security performance of the encryption algorithm in this paper, the encryption efficiency is also an important index in practice. Table 11 lists the time required to encrypt the two images “Lena” and “Cameraman” using the encryption algorithm proposed in this paper and the time required to encrypt images of the same size by other literature algorithms. We can see from the table that, compared to other encryption algorithms, our algorithm can better meet the needs of fast encryption.

### 5.8. Algorithm Comparative Analysis

In order to prove that the encryption algorithm in this paper has certain advantages, this paper compares and analyzes the security performance indicators of related literatures, and the comparison results are shown in Table 9 and Table 10. It is not difficult to see from Table 12 and Table 13 that the encryption algorithm in this paper has a good encryption effect and has certain advantages compared with the algorithms in the current advanced literature.

## 6. Conclusions

This paper proposes a new image encryption algorithm based on improved Arnold transform and a chaotic pulse-coupled neural network. First, the chaotic sequence is generated by the chaotic pulse-coupling neural network proposed in this paper, then the chaotic sequence is XORed with the pre-encrypted image to achieve the effect of pre-encryption, and then the image is scrambled by the improved Arnold transform to obtain the final encrypted image. Among them, the original Arnold transform is improved to better solve the limitation that the Arnold transform is only suitable for square images. At the same time, the pixel position index is introduced to eliminate the periodicity of Arnold transform, which improves the security of the Arnold transform for image scrambling, and it is not easy for illegal persons to use the periodic characteristics of Arnold transform to decipher.

Finally, experiments show that the pixel histogram of the encrypted image obtained by the algorithm in this paper is relatively uniform, which can break the statistical feature information of the original image and has the ability to resist statistical analysis attacks; the correlation coefficient of the encrypted image is close to 0, which can break the correlation between pixels in the original image; the information entropy is close to 8, which can resist statistical attacks. It has a large enough key space and can effectively resist brute force attacks; at the same time, the algorithm in this paper also has good resistance to differential attacks, anti-noise attack and anti-shearing attack ability, good robustness, and high security. Therefore, the algorithm in this paper has a good encryption effect.

## Figures and Tables

**Table 1:** The period of the standard Arnold transform.

Image Size N	Scrambling Period T	Image Size N	Scrambling Period T
10	30	32	24
14	24	64	48
16	12	128	96
18	12	256	192
25	50	512	384

**Table 2:** Histogram $\chi^{2}$ distribution statistic.

Image (256 × 256)	Original Original $\chi^{2}$	Encrypted Image $\chi^{2}$	Result
Lena	4.2981 × 10^5^	232.6328	**pass**
Cameraman	1.5196 × 10^6^	211.2109	**pass**
White	1.6712 × 10^7^	209.8203	**pass**
Black	1.6711 × 10^7^	259.1016	**pass**
Peppers	3.1639 × 10^4^	246.3125	**pass**
Plane	1.7322 × 10^5^	219.4922	**pass**

**Table 3:** Image adjacent pixel correlation coefficient.

Image (256 × 256)	Correlation Coefficient					
	Unencrypted			Encrypted		
	Horizontal	Vertical	Diagonal	Horizontal	Vertical	Diagonal
Lena	0.9204	0.9546	0.8944	0.0035	5.3876 × 10^−4^	3.2753 × 10^−4^
Cameraman	0.9756	0.9851	0.9601	−0.0020	0.0016	0.0013
White	--	--	--	0.0037	−7.5285 × 10^−4^	−7.6476 × 10^−4^
Black	--	--	--	−1.4710 × 10^−5^	0.0065	−0.0042
Peppers	0.9648	0.9697	0.9388	0.0015	0.0029	0.0019
Plane	0.9387	0.9320	0.8832	0.0018	−0.0057	−1.1486 × 10^−4^

**Table 4:** Image information entropy.

Image (256 × 256)	Information Entropy	
	Original	Encrypted
Lena	7.7758	7.9974
Cameraman	6.9749	7.9977
White	0	7.9977
Black	0	7.9972
Peppers	7.5798	7.9973
Plane	6.7334	7.9976

**Table 5:** Test results of TestU01.

Battery	Parameters	Number of Statistics	Result
SmallCrush	Standard	15	Pass
Alphabit	Standard	17	Pass
Rabbit	Standard	40	Pass
FIPS_140_2	Standard	16	Pass
BlockAlphabit	Standard	17	Pass

**Table 6:** Result of GVD score.

Image	GVD Score	Image	GVD Score
Lena	0.9415	Peppers	0.9674
Cameraman	0.9700	Plane	0.9512
White	1.000	Black	1.000

**Table 7:** Comparison results of key space.

Method	Year	Key Space
C.H. et al. [26]	2018	2^106^
G.D. et al. [27]	2018	2^186^
R.Z. et al. [28]	2019	2^199^
Xiaohong et al. [7]	2021	2^212^
Xw et al. [29]	2021	2^100^
Xiang H et al. [30]	2021	2^128^
Khalil Noura et al. [6]	2021	2^262^
Wang X et al. [4]	2021	2^420^
Our algorithm	**2022**	**2^430^**

**Table 8:** Key sensitivity analysis (%).

Image (256 × 256)	Initial Value	NPCR	UACI	Different Pixel Proportions
Lena	$a_{F}{\;+\;10}^{-10}$	99.6368	33.3494	99.64
	$V_{Em}{\;+\;10}^{-10}$	99.6170	33.4318	99.62
	$S_{ij}{\;+\;10}^{-10}$	99.6002	33.3646	99.60
	$a_{E}{\;+\;10}^{-10}$	99.6063	33.5540	99.61
	$x_{0}{\;+\;10}^{-10}$	99.6231	33.3861	99.62
Cameraman	$a_{F}{\;+\;10}^{-10}$	99.6445	33.3839	99.64
	$V_{Em}{\;+\;10}^{-10}$	99.5956	33.5159	99.60
	$S_{ij}{\;+\;10}^{-10}$	99.6078	33.4547	99.61
	$a_{E}{\;+\;10}^{-10}$	99.6109	33.4457	99.61
	$x_{0}{\;+\;10}^{-10}$	99.6445	33.4119	99.64
White	$a_{F}{\;+\;10}^{-10}$	99.6429	33.6027	99.64
	$V_{Em}{\;+\;10}^{-10}$	99.6689	33.5871	99.67
	$S_{ij}{\;+\;10}^{-10}$	99.5865	33.5613	99.59
	$a_{E}{\;+\;10}^{-10}$	99.6262	33.5986	99.63
	$x_{0}{\;+\;10}^{-10}$	99.5895	33.2934	99.62
Black	$a_{F}{\;+\;10}^{-10}$	99.6078	33.6249	99.61
	$V_{Em}{\;+\;10}^{-10}$	99.6063	33.5503	99.61
	$S_{ij}{\;+\;10}^{-10}$	99.5712	33.4333	99.57
	$a_{E}{\;+\;10}^{-10}$	99.6048	33.4588	99.60
	$x_{0}{\;+\;10}^{-10}$	99.5895	33.4558	99.59
**Average**	**--**	**99.614015**	**33.4841**	**99.6145**

**Table 9:** Comparison of different pixels of encrypted images before and after differential processing (%).

Image	Different Pixel Proportions	Image	Different Pixel Proportions
Lena	99.65	Cameraman	99.59
White	99.62	Black	99.58
Peppers	99.65	Plane	99.61

**Table 10:** Slight changes in the original image NPCR, UACI value (%).

Original Image (256 × 256)	NPCR	UACI
Lena	99.6460	33.4397
Cameraman	99.5880	33.5050
White	99.6170	33.4276
Black	99.5758	33.5291
Peppers	99.6506	33.4559
Plane	99.6063	33.4554
**Average**	**99.61395**	**33.468783**
**Ideal value**	**99.609375**	**33.463542**

**Table 11:** Encryption time comparison.

Image	Year	Image Size	Time (s)
Lena (Our method)	2022	256 × 256	0.1690
Cameraman (Our method)	2022	256 × 256	0.1740
JinLong et al. [36]	2021	256 × 256	0.6563
Wang et al. [37]	2021	256 × 256	0.2523
Wenying Wen et al. [38]	2020	256 × 256	2.1328
Farah M et al. [39]	2020	256 × 256	1.1202
Lena (Our method)	2022	512 × 512	0.7080
Cameraman (Our method)	2022	512 × 512	0.6640
Wenying Wen et al. [38]	2020	512 × 512	18.1354
José, A. et al. [40]	2019	512 × 512	10.4200
Lena (Our method)	2022	1024 × 1024	2.2990
Cameraman (Our method)	2022	1024 × 1024	2.1700

**Table 12:** Performance comparison of different encryption algorithms.

Image (256 × 256)	Method	Year	Info Entropy	Correlation Coefficient		
				Horizontal	Vertical	Diagonal
Lena	Li et al. [41]	2020	7.9894	0.0044	0.0015	0.0019
	Wang et al. [42]	2020	7.9969	0.0006	0.0082	0.0032
	Kamrani et al. [43]	2020	7.9945	--	--	--
	Hosny et al. [22]	2021	7.9972	0.0069	0.0479	0.0075
	Xw et al. [29]	2021	7.9971	−0.0017	−0.0132	0.0084
	Zhang et al. [44]	2021	7.9969	0.0040	−0.0012	−0.0021
	Farhan et al. [45]	2021	7.9971	−0.0004	−0.0028	0.0040
	Wang et al. [37]	2021	7.9960	0.0023	0.0020	0.0073
	Xiang et al. [30]	2021	7.9972	0.0013	-0.0041	−0.0044
	JinLong et al. [36]	2021	7.9858	0.0031	0.0076	−0.0026
	Proposed	2022	7.9974	−0.0035	5.3876 × 10^−4^	3.2753 × 10^−4^
Cameraman	Niu et al. [46]	2020	7.9971	−0.0070	0.0083	0.0013
	Kamrani et al. [43]	2020	7.9947	--	--	--
	Wu et al. [47]	2021	7.9935	−0.0036	0.0048	0.0073
	JinLong et al. [36]	2021	7.9868	−0.0252	−0.0060	−0.0078
	Proposed	2022	7.9977	0.0020	0.0016	0.0013
Peppers	Hua, Z. et al. [48]	2019	7.9971	0.0196	0.0165	0.0210
	Minjun et al. [49]	2020	7.9970	0.00476	−0.009531	0.007338
	Wang et al. [37]	2021	7.9964	−0.0037	0.0035	−0.0057
	Xw et al. [29]	2021	7.9971	−0.0062	−0.0236	−0.0047
	Wu et al. [47]	2021	7.9941	−0.0170	−0.0334	−0.0073
	Hosny et al. [22]	2021	7.9970	0.0211	0.0129	0.0013
	Proposed	2022	7.9973	0.0015	0.0029	−0.0019
Plane	Wu et al. [50]	2018	7.9970	0.0028	0.0041	0.0010
	Hua, Z. et al. [48]	2019	7.9971	0.0055	0.0014	0.0083
	Xw et al. [29]	2021	7.9972	−0.0043	−0.0236	−0.0047
	Hosny et al. [22]	2021	7.9972	0.0229	0.0103	0.0100
	Wang et al. [37]	2021	7.9959	0.0054	0.0027	0.0028
	Proposed	2022	7.9976	−0.0018	−0.0057	−1.1486 × 10^−4^

**Table 13:** Performance comparison of different encryption algorithms.

Image (256 × 256)	Method	Year	NPCR (%)	UACI (%)	$\chi^{2}$
Ideal value			99.609375	33.463542	Minimum
Lena	Kamrani et al. [43]	2020	99.7864	30.3256	--
	Li et al. [41]	2020	99.66	33.42	--
	Minjun et al. [49]	2020	99.6114	33.4523	--
	Hosny et al. [22]	2021	99.6246	33.4226	264.8750
	Zhang et al. [44]	2021	99.62	33.50	--
	Wang et al. [37]	2021	99.5894	33.4629	--
	Xw et al. [29]	2021	--	--	266.6797
	Proposed	2022	99.6460	33.4397	232.6328
Cameraman	Kamrani et al. [43]	2020	99.791	27.6376	--
	Zhang et al. [44]	2021	99.63	33.56	--
	Wang et al. [37]	2021	99.5879	33.4553	--
	Proposed	2022	99.5880	33.5050	211.2109
Peppers	Minjun et al. [49]	2020	99.6115	33.4245	--
	Hosny et al. [22]	2021	99.6033	33.4274	268.4766
	Xw et al. [29]	2021	--	--	260.3906
	Proposed	2022	99.6506	33.4559	246.3125
Plane	Minjun et al. [49]	2020	99.6043	33.2875	--
	Xw et al. [29]	2021	--	--	252.1172
	Proposed	2022	99.6063	33.4554	219.4922

## Data Availability

Not applicable.
